# Genome and transcriptome sequencing of the halophilic fungus *Wallemia ichthyophaga*: haloadaptations present and absent

**DOI:** 10.1186/1471-2164-14-617

**Published:** 2013-09-13

**Authors:** Janja Zajc, Yongfeng Liu, Wenkui Dai, Zhenyu Yang, Jingzhi Hu, Cene Gostinčar, Nina Gunde-Cimerman

**Affiliations:** 1 Department of Biology, Biotechnical Faculty, University of Ljubljana, Večna pot 111, SI-1000 Ljubljana, Slovenia; 2 BGI-Shenzhen, Main Building 11/F, Beishan Industrial Zone, Yantian District, Shenzhen 518083, China; 3 Centre of Excellence for Integrated Approaches in Chemistry and Biology of Proteins (CIPKeBiP), Jamova 39, SI-1000 Ljubljana, Slovenia

**Keywords:** *Wallemia ichthyophaga*, Wallemiomycetes, Genome, Transcriptome, Phylogeny, Haloadaptation, Halotolerance, Hypersaline, Extremophile, Hydrophobin

## Abstract

**Background:**

The basidomycete *Wallemia ichthyophaga* from the phylogenetically distinct class Wallemiomycetes is the most halophilic fungus known to date. It requires at least 10% NaCl and thrives in saturated salt solution. To investigate the genomic basis of this exceptional phenotype, we obtained a *de-novo* genome sequence of the species type-strain and analysed its transcriptomic response to conditions close to the limits of its lower and upper salinity range.

**Results:**

The unusually compact genome is 9.6 Mb large and contains 1.67% repetitive sequences. Only 4884 predicted protein coding genes cover almost three quarters of the sequence. Of 639 differentially expressed genes, two thirds are more expressed at lower salinity. Phylogenomic analysis based on the largest dataset used to date (whole proteomes) positions Wallemiomycetes as a 250-million-year-old sister group of Agaricomycotina. Contrary to the closely related species *Wallemia sebi*, *W. ichthyophaga* appears to have lost the ability for sexual reproduction. Several protein families are significantly expanded or contracted in the genome. Among these, there are the P-type ATPase cation transporters, but not the sodium/ hydrogen exchanger family. Transcription of all but three cation transporters is not salt dependent. The analysis also reveals a significant enrichment in hydrophobins, which are cell-wall proteins with multiple cellular functions. Half of these are differentially expressed, and most contain an unusually large number of acidic amino acids. This discovery is of particular interest due to the numerous applications of hydrophobines from other fungi in industry, pharmaceutics and medicine.

**Conclusions:**

*W. ichthyophaga* is an extremophilic specialist that shows only low levels of adaptability and genetic recombination. This is reflected in the characteristics of its genome and its transcriptomic response to salt. No unusual traits were observed in common salt-tolerance mechanisms, such as transport of inorganic ions or synthesis of compatible solutes. Instead, various data indicate a role of the cell wall of *W. ichthyophaga* in its response to salt. Availability of the genomic sequence is expected to facilitate further research into this unique species, and shed more light on adaptations that allow it to thrive in conditions lethal to most other eukaryotes.

## Background

*Wallemia* Johan-Olsen (Wallemiales, Wallemiomycetes) is a genus of cosmopolitan xerophilic fungi that are found in a wide variety of environments characterised by low water activity (a_w_) [1,2]. According to the characterisation of dolipore septa [3,4] and to molecular analysis [2,5], *Wallemia* was placed in the phylum Basidiomycota. Through various studies, its inferred phylogenetic origin varied from the root of basidiomycetes [2], to *incertae sedis*[6], to being a sister group of the Agaricomycotina and Ustilaginomycotina [5] or only of the Agaricomycotina [4]. Previously, the genus contained only one species, but it was later segregated into three species: *Wallemia ichthyophaga*, *Wallemia sebi* and *Wallemia muriae*[2].

To date, only a limited number of strains of *W. ichthyophaga* have been isolated from hypersaline water of solar salterns, bitterns (magnesium-rich residual solutions in salt production from sea water) and salted meat (ham: *prosciutto*) [2] (Sonjak et al., unpublished data). In addition to differences in phylogenetic DNA markers, *W. ichthyophaga* is also distinguished from the other two representatives of the genus by its characteristic morphology and halophilic physiology [2,7]. Although xerotolerance is rare in the Basidiomycota, all three *Wallemia* spp. are among the most xerophilic fungal taxa [2]. However, while *W. sebi* and *W. muriae* strongly prefer high concentrations of non-ionic solutes (for example sugars) over those of NaCl (although they can also tolerate up to 4.6 M and 4.3 M NaCl, respectively [8]), the opposite is true for *W. ichthyophaga*[2]. *W. ichthyophaga* requires at least 1.5 M NaCl for *in-vitro* growth (or some other osmolyte for an equivalent a_w_), and it even thrives in saturated NaCl solution. It also tolerates high concentrations of other salts, such as MgCl_2_ (Sonjak et al., unpublished data). Such a narrow ecological amplitude is common for specialised archaeal halophiles, but in the fungal kingdom it is an exception. Even the most salt-tolerant fungal species do not normally require salt for growth, and they frequently have their growth optimum in the absence of salt. Because of this, *W. ichthyophaga* is a rare fungal example of an obligate extremophilic specialist [9], and it is considered to be the most halophilic fungus known to date.

Studies of haloadaptation mechanisms of *W. ichthyophaga* began relatively recently and are thus still at early stages. The fungus counterbalances the osmotic pressure caused by high concentrations of salt in the surrounding medium by intracellular accumulation of a mixture of polyols, among which glycerol is the major osmotically regulated solute (Zajc et al., unpublished data). It was previously published that *W. ichthyophaga* has a glycerol-3-phosphate dehydrogenase gene (*WiGPD1*) that encodes the key enzyme in the biosynthesis of glycerol. Its expression elevated at high concentrations of salt. Comparisons of Gpd1 from the salt-sensitive *Saccharomyces cerevisiae* to WiGpd1 have shown high overall amino-acid similarity; however, *WiGPD1* lacks the N-terminal peroxisomal targeting (PTS2) sequence, which is important for its peroxisome localisation [10,11]. The consequent constant cytosolic localisation of Gpd1 might thus be beneficial for organisms that live in extremely saline environments [11].

High-osmolarity glycerol (HOG) signalling pathway in fungi is responsible for the sensing of osmolarity changes and for the facilitation of adaptation of cells to hypersaline environment in *S. cerevisiae*[12]. This is also the case in the extremely halotolerant black yeast *Hortaea werneckii*[13]. Several, but not all, of the genes of this pathway have been found in the genome of *W. sebi*[4]. In *W. ichthyophaga* the homologues of MAP kinases Hog1 have recently been studied in detail [14]. Two homologues were found (WiHog1A and WiHog1B), but only one of them was able to complement the *hog1Δ* strain of *S. cerevisiae* and activate the HOG-responsive glycerol-3-phosphate dehydrogenase (GPD) promoter. The transcription of both genes was lowest at optimal salinity (20% NaCl), while at limiting salinities (10% and 30% NaCl) the transcription increased by at least 2- and up to 6-fold. The proteins were dephosphorylated after exposing the cells to both hypo- and hyper-osmotic shocks, a pattern opposite to that of *S. cerevisiae*[14].

High concentrations of salt trigger substantial morphological changes to *W. ichthyophaga* cells. These are believed to have important adaptive roles under hypersaline conditions. This fungus grows in the form of sarcina-like structures, or compact multicellular clumps [2]. This morphology can be observed in several phylogenetically distant polyextremotolerant species, and it is believed to enhance survival in high-stress environments [15-17]. The cells have an abundant cover of extracellular polysaccharides [8], which have been reported to protect against desiccation in rock-inhabiting fungi [18], and which might also have a protective role at high salinity. Apart from an almost four-fold increase in the size of cell clumps, the most striking morphological response of *W. ichthyophaga* to high salinity is a three-fold thickening of the cell wall, which results in a substantially decreased functional cell volume [8].

In environments such as salterns, which are believed to be the primary habitat of *W. ichthyophaga*, the levels of toxic sodium ions (Na^+^) far exceed those of potassium ions (K^+^). Under these conditions, the cells must use a lot of energy on active transmembrane transport of ions, to maintain a stable and high intracellular K^+^/Na^+^ ratio. This is achieved by transporters that have higher affinity for K^+^ than for Na^+^ at the level of influx, by efficient efflux of toxic or surplus cations from the cells, and also by selective compartmentalisation of cations in organelles (reviewed in [19]). The transport systems that mediate these alkali-metal cation fluxes at both the plasma and organelle membranes function together not only to maintain K^+^ homeostasis and to eliminate toxic Na^+^ (or Li^+^), but also to preserve membrane potential, regulate intracellular pH, and maintain positive turgor inside the cell, which is necessary for plasma-membrane/ cell-wall expansion and cell division and to cope with osmotic stress (reviewed in [19]). Hence, the alkali-metal cation influx and efflux systems of these halotolerant eukaryotes are of great interest for the explanation of osmoadaptation to extremely saline environments. Nevertheless, no studies that focus on *W. ichthyophaga* ion transporters have been published to date*.*

The mating behaviour of the *Wallemia* spp. is also unclear. To date, no teleomorphs or fruiting bodies have been observed in any of the *Wallemia* spp. The existence of a single mating type locus and an almost complete set of meiosis genes encoded in the genome of *W. sebi* suggest the capability for sexual reproduction [4]. However, genetic evidence for sexual reproduction in *W. ichthyophaga* has not yet been assessed.

Our knowledge of the mechanisms underlying the exceptional ability of *W. ichthyophaga* to thrive at salt concentrations that are lethal to the vast majority of eukaryotes and all but the most adapted prokaryotes is only starting to expand. This is partly due to the relatively recent taxonomic description of this species, although it is also a consequence of the significant experimental input needed for even the most basic discoveries. To alleviate this problem and to facilitate further work with *W. ichthyophaga*, results of *de-novo* sequencing of its whole genome and transcriptomes of the cells grown at two limiting salinities are presented here. The characteristics of the genome and the predicted proteome and transcriptomes are described and discussed in light of the halophilic nature of *W. ichthyophaga*, together with the apparent inability for sexual reproduction of this species. The phylogenetic position of *Wallemiomycetes* is determined with excellent support values on the basis of the whole proteome, which is the largest dataset used for this purpose, so far.

## Results and discussion

### Genome sequencing, assembly and gene prediction

**Figure 1:**
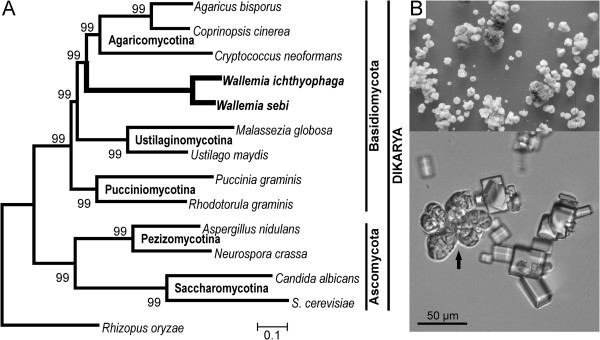
***Wallemia ichthyophaga *. A**. Phylogram showing the phylogenetic origin of *W. ichthyophaga*, inferred from a super alignment of selected fungal proteomes. Chi2-based branch supports are shown, calculated according to the approximate Likelihood-Ratio Test, as implemented in Phyml 3.0. **B**. Colonies of *W. ichthyophaga* on yeast nitrogen base medium with 25% NaCl (w/v), and a microscopic image showing the characteristic meristematic clumps (arrow) formed by isodiametric growth of groups of thick-walled cells next to the cubic crystals of halite (NaCl).

**Figure 2:**
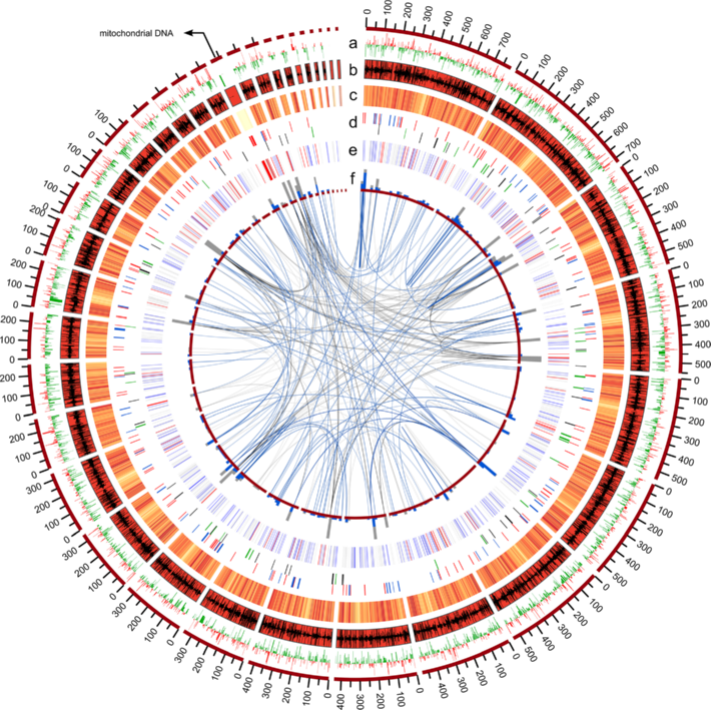
**Circular representation of the *Wallemia ichthyophaga *genome.** The following data are shown (from outside, in): **(a)** Differential expression as log_2_ ratio of expression at high salinity (30% NaCl [w/v]) *versus* low salinity (10% NaCl [w/v]), with increased expression in red and decreased in green (scale −2 to 2); genes with false discovery rates larger than 0.001 are not shown. **(b)** Sizes of scaffolds >10 kbp, embedded is a histogram of RPKM values (the number of reads which map per kilobase of the exon model per million mapped reads), with expression at high salinity oriented outwards, and low salinity oriented inwards. **(c)** GC content in 1 kbp windows on a scale from 30% (yellow) to 60% (red). **(d)** Locations of certain groups of genes (red, energy production; blue, cell cycle; black, cell wall; green, membrane transporters). **(e)** Locations of repetitive sequences (grey, tandem repeats; blue, transposons; red tRNA). **(f)** Gene duplications and links, linking their locations determined by aligning the predicted proteins to the genome with Exonerate (cut-off: at least 50% of maximum score obtainable for each query). Blue, proteins that aligned with more than 200 amino acids; grey, proteins that aligned with 100–200 amino acids.

This Whole Genome Shotgun project has been deposited at DDBJ/EMBL/GenBank under the accession [GenBank:APLC00000000]. The version described in this paper is the first version, [GenBank:APLC01000000]. For all genes and proteins discussed here, GenBank accession numbers are provided in the text. The total assembly size of the *W. ichthyophaga* genome is 9.6 Mb, and it is assembled into 101 contigs and 82 scaffolds (Table 1). This genome size is even smaller than for the closely related species *W. sebi* (9.8 Mb; Figure 1, Table 1) [4]. While even smaller genomes exist (*Malassezia globosa* at 9.0 Mb), most basidiomycetous haploid genomes are at least twice as large, and range even up to more then 40-times larger [20]. In line with its small size, the genome of *W. ichthyophaga* is very compact. It contains only 1.67% repetitive sequences, and almost three quarters of the genome is covered by the coding DNA sequences. The overall GC content is 45.35%, and it is characteristically lower in the mitochondrial DNA (Figure 2). While relatively low, this value is still higher than *W. sebi*, which contains only 40.01% GC. The gene density is 514 genes/ Mb scaffold, which is higher than in *M. globosa* (476 genes/ Mb) and only slightly lower than *W. sebi* (538 genes/ Mb). The absolute number of predicted proteins (4884) is also unusually small and in the range observed for *Escherichia coli*[21]. For *W. sebi* 5284 proteins were predicted, and 4285 for *M. globosa*, while >10000 proteins are not uncommon in other basidomycetes). Despite the reduction in genome size and gene number the number of introns is not unusually small as is seen in some other fungi with small genomes [22]: on average, the predicted genes contain 2.41 introns that are 61 bp long (Table 1). In other fungi the average intron densities range from just over 1.0 intron/ kb coding sequence (cds) in *Schizosaccharomyces pombe* to approximately 5.0 introns/ kb cds in *Cryptococcus neoformans*[23]. For all of the predicted proteins, 3715 had hits in the SwissProt database (e-value cut-off, 10^-6^), 3278 genes were mapped in the Kyoto Encyclopaedia of Genes and Genomes (KEGG) database, and 2603 were classified in the Clusters of Orthologous Groups (COG) database (Additional file 1: Figures S1 and S2).

**Table 1:** ***Wallemia ichthyophaga *(EXF-994) genome assembly statistics and comparison with *Wallemia sebi *(adapted from **[4]** or calculated from the genomic data published online **[76]**)**

**Statistic**	***W. ichthyophaga***	***W. sebi***
Coverage	>270×	71×
Genome assembly size (Mb)	9.625	9.82
Number of scaffolds	82	56
Scaffold N50 (Mb)	0.44	0.34
Number of contigs	101	114
Contig N50 (Mb)	0.35	
CDS total length (Mb)	7.083 (73.59% of genome)	6.701 (68.39% of genome)
CDS average size (bp)	1450	1268
Predicted protein-coding genes (n)	4884	5284
Predicted proteins, average length (aa)	483	423
Exon total length (Mb)	7.083	
Exon total number	16670	
Exon average length (bp)	424	410
Introne total length (Mb)	0.719	
Introne total number	11786	
Introne average length (bp)	61	55
GC content (%)	45.35	40.01
GC content of CDS (%)	47.51	42.06
Repeat content (kb)	160.929 (1.67% of genome)	
- Tandem repeats (kb)	85.47	
- DNA transposons (kb)	84.77	
tRNA (kb)	11.55	

**Figure 3:**
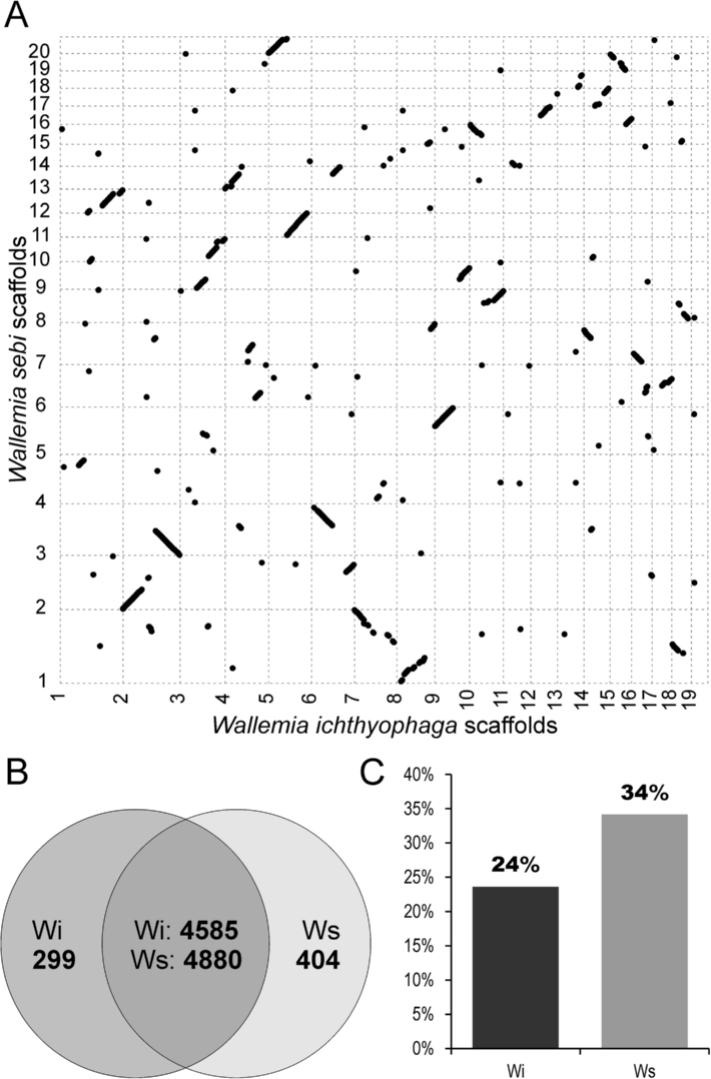
**Genomic and proteomic comparison of *Wallemia ichthyophaga *(Wi) and *Wallemia sebi *(Ws). A.** Dot-plot comparison of scaffolds longer than 200 kbp. The six-frame translations of scaffolds were aligned with Mummer 3.23. Homologous regions are plotted as dots. Scaffolds of each species are displayed ordered by decreasing size along x and y axes. Diagonal lines of dots in specific boxes represent syntenic regions. **B.** Shared and unique proteins of *W. ichthyophaga* and *W. sebi*, as determined by all-against-all blast (e-value cut-off, 10^-6^). **C.** Proportion of unique proteins that were matched to at least one Pfam family.

Alignment of the whole genomic sequence with the genome of *W. sebi* reveals long syntenic regions (Figure 3A). According to BLASTp analysis (e-value cut-off, 10^-6^), 93.9% of the proteins from *W. ichthyophaga* have homologues in the proteome of *W. sebi* (Figure 3B). Among unique proteins, only a quarter can be classified into at least one protein family, according to the Pfam database (Figure 3C). The large overlap between these species is not surprising given their close phylogenetic proximity. Both of these fungi were originally classified as *W. sebi*, until this species was segregated into *W. ichthyophaga*, *W. sebi* and *W. muriae*, based on differences in conidial size, xerotolerance, and sequence data [2]. Proteins present in *W. ichthyophaga* but absent in *W. sebi* include several proteins related to DNA processing and DNA damage: two 5’-3’ exoribonucleases [GenBank:EOR04230, GenBank:EOQ98776]; a telomerase reverse transcriptase [GenBank:EOR00183]; a DNA repair protein RAD50 [GenBank:EOR01216] and a DNA polymerase similar to mu polymerase [GenBank:EOR02079], both of which are involved in non-homologous end joining repair; an ATP-dependent DNA helicase [GenBank:EOR02849]; and a DNA damage-inducible protein 1 [GenBank:EOR00413]. Non-homologous recombination may offer *W. ichthyophaga* an additional mode of mitotic recombination, which would be important in light of the fact that this species (contrary to *W. sebi*) appears to be incapable of meiosis and sexual reproduction, as discussed below.

### Phylogenetic position of Wallemiomycetes

The phylogenetic position of Wallemiomycetes in published studies has been determined as at the base of Basidiomycota, as a sister group to either Agaricomycotina or Ustilaginomycotina or both, or simply as *incertae sedis* within Basidiomycota [2,4-6]. However, these analyses used only limited sets of genes or proteins. In the present study, the whole proteomes of 14 fungi were aligned, and the resulting phylogeny resolved the position of the *Wallemia* spp. as a sister group to (or the earliest diverging lineage of) Agaricomycotina (Figure 1). This also supports Pucciniomycotina as the earliest diverging lineage within the Basidiomycota analysed. These results are in agreement with phylogenetic positioning of *W. sebi* published by Padamsee et al. [4].

Previously published calibration points were used to construct the chronogram [24]: *Rhizopus oryzae*–Dykaria split 495 million years ago (mya), Ascomycota–Basidiomycota split 452 mya, Pezizomycotina crown 215 mya, Basidomycota crown 340 mya. Under these assumptions, the split between *W. ichthyophaga* and *W. sebi* is estimated as 11.9 mya, and that between Wallemiomycetes and Agaricomycotina as 250 mya. As the calibration of the fungal tree of life remains uncertain due to scarcity and poor preservation of fossil material, these values can only be considered as rough estimates.

### Expansions and contractions of the protein families

**Figure 4:**
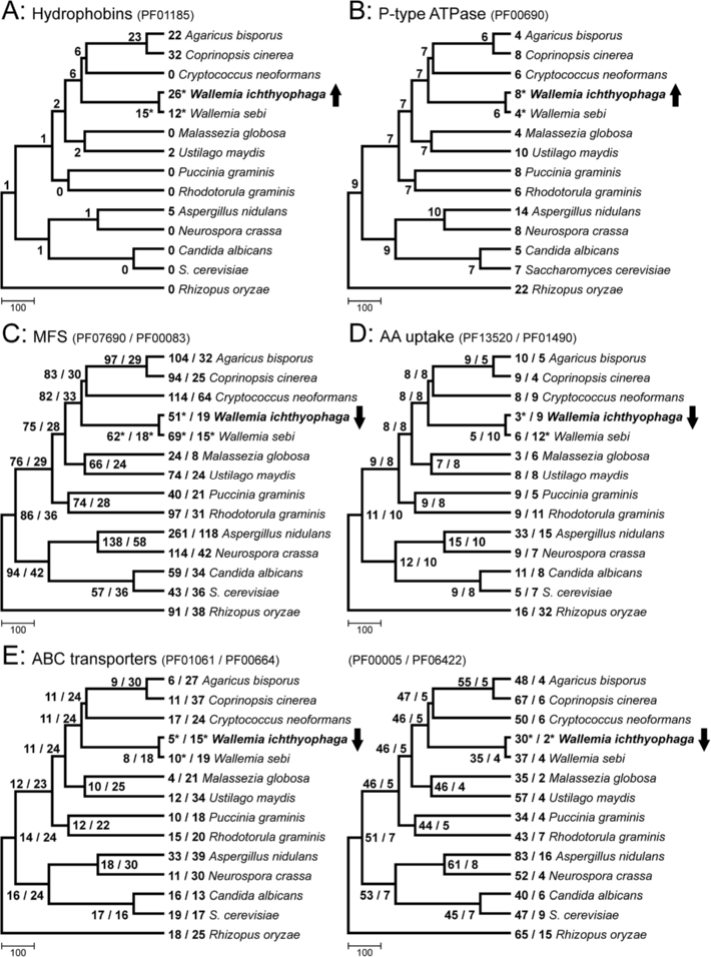
**Extension and contraction of selected protein families.** Proteins were classified into families according to the Pfam database, and the number of representatives in each family was analysed with the CAFE software. All families shown were significantly expanded (upward arrow) or contracted (downward arrow) in the proteome of *Wallemia ichthyophaga.* The numbers of representatives of each protein family in the contemporary species as well as the estimated ancestral states are shown in the trees. Statistically significant numbers for *W. ichthyophaga*, *W. sebi* and their last common ancestor are marked with an asterisk. The chronogram of analysed species was reconstructed on the basis of whole proteomes, and calibrated according to previously published calibration points in the fungal tree of life (scale unit, millions of years). **A**. Protein family of fungal hydrophobins (PF01185). **B**. Protein family of cation transporting (P-type) ATPases (PF00690). **C**. Major facilitator superfamily (MFS) and sugar transporters (PF07690, PF00083). **D**. Protein families of amino-acid permeases and transporters (AA uptake; PF13520, PF01490). **E**. Protein families of ATP binding cassette (ABC) transporters (PF01061, PF00664, PF00005, PF06422).

Of the predicted proteins, 3924 (80.3%) contain at least one of the 2678 Pfam domains in *W. ichthyophaga* (Additional file 2: Table S1). Among the families represented by the most proteins, there are several connected to transport functions in the cell: e.g., major facilitator family (PF07690), mitochondrial carriers (PF00153), ABC transporters (PF00005, PF00664) and others (PF00083). Seven protein families are significantly expanded and 19 are contracted in *W. ichthyophaga*, while 17 are significantly changed in the predicted last common ancestor of *W. ichthyophaga* and *W. sebi* (Additional file 3: Table S2). Among the proteins that are enriched, there are hydrophobins (PF01185; Figure 4A) and P-type ATPases (PF00690; Figure 4B).

### Hydrophobins

The hydrophobins are cell-wall proteins that are secreted by filamentous fungi and have roles in a broad range of processes in growth and development [25]. Hydrophobins are characterised by their small size (≤20 kDa) and amphipathic nature, with their hydrophobic and hydrophilic domains [26]. There are multiple hydrophobin genes in the genome of individual fungi, due to possibly different functional roles or differential expression, or to different environmental conditions or developmental stages. In the predicted last common ancestor of *W. ichthyophaga* and *W. sebi*, 15 hydrophobins are estimated. A significant enrichment of the hydrophobin protein family occurred in *W. ichthyophaga* (to 26 representatives), and on the contrary, a significant contraction occurred in *W. sebi* (to 12 representatives; Figure 4A). Among all of the protein family expansions in *W. ichthopyhaga*, this is the most significant.

**Figure 5:**
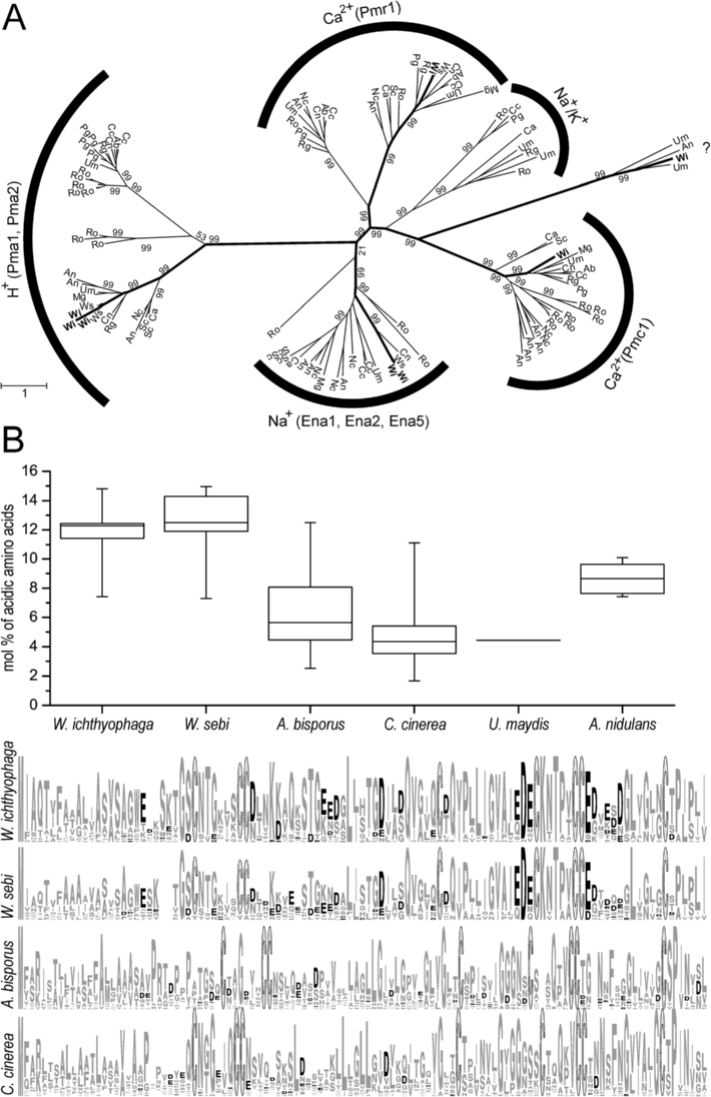
**Analysis of significantly extended protein families. A.** Gene tree of proteins with the Pfam domain PF00690 (P-type ATPase transmembrane transporters). The tree was constructed with the PhyML 3.0 software. Chi2-based approximate Likelihood-Ratio Test branch supports are shown for the major groups. Abbreviations of fungal species from which the proteins originate: Ab, *Agaricus bisporus*; An, *Aspergillus nidulans*; Ca, *Candida albicans*; Cc, *Coprinopsis cinerea*; Cn, *Cryptococcus neoformans* var. *grubii*; Mg, *Malassezia globosa*; Nc, *Neurospora crassa*; Pg, *Puccinia graminis*; Ro, *Rhizopus oryzae*; Rg, *Rhodotorula graminis*; Sc, *Saccharomyces cerevisiae*; Um, *Ustilago maydis*; Wi, *Wallemia ichthyophaga*; Ws, *Wallemia sebi*. The groups are labelled with the cations that are transported by the given group of ATPases, and the names of the *S. cerevisiae* proteins in that group (in brackets). **B.** Top: Proportion of acidic amino acids in hydrophobins from various fungal species. Box chart of quartiles shows the molar pecentages of acidic amino acids in individual hydrophobin proteins, with minima and maxima shown by the whiskers. *Ustilago maydis* contains only one hydrophobin of expected length. Bottom: The graphical representation of multiple sequence alignment of all hydrophobins from a given species shows the conservation of individual positions and amino acids. Black, acidic amino acids; light grey lined with black, cysteine residues; grey, all other residues.

As previously shown [26], and as can be seen from our multiple sequence alignment of all of the hydrophobins from the list of basidiomycetous species analysed (Figure 5B, bottom), the DNA sequence similarities for the different hydrophobins are usually low between the different species. The most important feature of the primary sequence, which is common to all hydrophobins, is the characteristic pattern of conserved spacing of eight cysteine residues that form four disulphide bridges [26,27]. These are also conserved in the hydrophobins of *W. ichthyophaga*. Interestingly, the hydrophobins of *W. ichthyophaga* and its closest relative *W. sebi* are similar, and they share a large fraction of conserved positions of their amino acids (Figure 5B, bottom). They also both contain a high proportion of acidic amino acids, as compared to other fungi (Figure 5B, top). Higher proportion of acidic amino-acids is characteristic of proteins exposed to high concentrations of salt as has been noted in halophilic proteins of Archaea [28]. As hydrophobins are likely to be directly exposed to the external high concentrations of NaCl in *W. ichthyophaga*, the unusually high proportion of acidic amino acids is not surprising. Acidic amino acids on a protein surface enable the binding of large amount of salts and water under solvent conditions, and in this way they maintain soluble and active conformations in an environment that is generally detrimental to other proteins [29]. Moreover, it has been shown that halophilic proteins are characterised by low hydrophobicity and under-representation of cysteines [30]. Therefore, it is interesting that halophilic hydrophobins have at least moderate levels of hydrophobicity and are cysteine rich, although, at the same time, they show salinity-biased amino-acid compositions.

The property of the hydrophobins to spontaneously assemble at hydrophobic–hydrophilic interfaces to form amphipathic monolayers governs their diverse functions in the growth and development of filamentous fungi. For instance, the hydrophobins allow these cells to breach the air–water interface, prevent water-logging while maintaining permeability to gaseous exchange, enable attachment to hydrophobic surfaces, modify the movement of solutes across the cell wall, and give strength and rigidity to the cell wall [25,31]. Some of these functions would also be beneficial in hypersaline environments. Modulation of cell wall permeability could be of great importance in an environment where toxic salt ions are constantly leaking into the cell, while strengthening and rigidifying the cell wall would be useful during changes of environmental osmolarity. Given that certain hydrophobins are responsible for microconidial chain formation in *Fusarium verticillioides*[32], hydrophobins in *W. ichthyophaga* might additionally be involved in the aggregation of these cells into compact clumps, as is characteristic of this fungus. Formation of meristematic clumps is observed as a stress-response in many unrelated halotolerant fungi [9]. Both the changes in the cell wall and the formation of multicellular structures are reported to be among the main adaptations of *W. ichthyophaga* to hypersaline environments [8]*.*

Over the last few years, the hydrophobins have received a lot of attention from biotechnologists. Their ability to reverse the hydrophilic–hydrophobic character of a surface and/or their surfactant capacity has many potential applications. They can be used as surfactants and emulsifiers in food processing, in anti-fouling coatings, surface coating of biomaterials, such as surgical instruments and medical implants, and immobilisation of various substances. They might even have a role as functional coatings of drug nanoparticles ([26,27,33]. The differences in the amino-acid compositions of the hydrophobins from the halophilic *W. ichthyophaga* (and especially the high number of acidic amino acids) might give these proteins unique properties. These are well worth investigating too, as they might expand the scenarios of hydrophobin use in novel applications or in known applications under specific conditions, such as those with high salt concentrations.

### Transporters of alkali metal cations

The significant enrichment in *W. ichthyophaga* of the protein family of cation-transporting ATPases (PF00690) might contribute to its halophilic ecotype. This protein family of cation proteins is represented by three H^+^ and two Na^+^ P-type ATPases in the plasma membrane, one of each of the two groups of Ca^2+^ P-type ATPases in vacuoles (Pmc1) and the Golgi apparatus (Pmr1), and a protein with unknown specificity (Figure 5A, Table 2).

**Table 2:** **Major plasma membrane and intracellular transporters of *Wallemia ichthyophaga *(Wi) and *Wallemia sebi *(Ws)**

**Cellular location**^**a**^	**Transporter type**	**Substrate specificity/ main function**	**Name of the Sc**^**b**^**homologue**	**Number of homologues in Wi**^**c**^	**Number of homologues in Ws**^**d**^
PM	Channel	K^+^ efflux	Tok1	0	0
	Uniporter	K^+^ uptake	Trk1,2	1	1
	P-type ATPase	Na^+^ (and Li^+^) efflux	Ena1,2,5	**2** ↓^c^	**1**
	Antiporter	Na^+^, (K^+^)/ H^+^ exchange	Nha1	2	2
	Antiporter	Na^+^/H^+^ exchange	/	1	1
	Symporter	Na^+^/P_i_ cotransporter	Pho89	1 ↑^d^	1
	P-type ATPase	H^+^ export	Pma1,2	**3**	**2**
unknown	Permease	Ca^2+^ permease	/	1	1
	Antiporter	Ca^2+^/H^+^ exchange	/	1	1
	P-type ATPase	cation transporting, unknown specificity	/	2 ↓^e^	2
GA	Antiporter	K^+^/H^+^ exchange	Kha1	2	2
	P-type ATPase	Ca^2+^ and Mn^2+^ transport into GA	Pmr1	1	1
LE	Antiporter	Na^+^, (K^+^)/ H^+^ exchange	Nhx1	1	1
	Antiporter	Na^+^, K^+^ /H^+^ exchange	Vnx1	1	1
VAC	V-type ATPase	H^+^ in vacuole	Vma1	1	1
	P-type ATPase	depleting cytosol of Ca^2+^ ions	Pmc1	1	1
MTH	Antiporter	K^+^/H^+^ exchange	Mrs7/Mdm38	1	1

^a^*PM* plasma membrane, *GA* Golgi apparatus, *LE* late endosomes, *VAC* vacuole, *MTH* mitochondria.

^b^Sc, *S. cerevisiae*; bold numbers mark differences in numbers of transporters between *W. ichthyophaga* and *W. sebi.*

^c,d,e^ Differential expression of the transporter homologues in *W. ichthyophaga* (log_2_ ratios: c: ↓ -1.00; d: ↑ +1.52; e: ↓ -1.27).

In the extensively studied model of yeast *S. cerevisiae*, the two H^+^-exporting P-type (Pma1, 2) and several Na^+^-exporting Ena ATPases (i.e., *exitus natru*: exit of Na^+^), are crucial for the maintenance of homeostasis of intracellular K^+^ and Na^+^[34]. In *S. cerevisiae*, Pma pumps are the most abundant plasma-membrane protein. These consume at least 20% of the cellular ATP [35], to generate the electrochemical gradient of H^+^ across the plasma membrane, which is indispensable for all secondary active symporters and antiporters [19]. The *W. ichthyophaga* genome encodes three putative Pma proton pumps [GenBank:EOR02126, GenBank:EOR02128, GenBank:EOR00565], which are highly similar to the only two Pma ATPases from *W. sebi* (over 91% identity).

The maintenance of low concentrations of toxic Na^+^ occurs via two types of Na^+^ efflux systems in the plasma membrane. Ena P-type ATPases couple the hydrolysis of ATP to the export of Na^+^ (or K^+^) against the electrochemical gradient, while Nha antiporters export Na^+^ by using the transmembrane H^+^ gradient. These systems have complementary functions: Ena ATPases are more important at high pH, which does not allow for the correct functioning of Nha antiporters. In *S. cerevisiae* the ENA cluster, particularly *ENA1*, is a major determinant of salt tolerance in this yeast (reviewed in [19]). The two Ena ATPases of *W. ichthyophaga* [GenBank:EOR00553, GenBank:EOR04078] are 92% identical and are particularly different from *ScENA1* (36% and 37% identity). Interestingly, in *W. sebi*, there is only one ENA ATPase.

P-type ATPases are not the only ones responsible for the maintenance of the cellular ion homeostasis. A variety of other secondary active transporters contribute to keeping the intracellular concentrations of highly toxic Na^+^ low, while at the same time maintaining a constant level of K^+^. These tasks are particularly problematic in the environments predominated by the high concentrations of Na^+^. By searching the genome of *W. ichthyophaga* for homologues of known transporters from *S. cerevisiae*[19] and unconventional yeast [36], we identified several plasma-membrane (Nha1, Trk1, Pho89, Ena and Pma) and intracellular (Kha1, Pmr1, Nhx1, Vnx1, Vma1, Pmc1, Mrs7/Mdm37) homologues, as summarised in Table 2. Apart from the above mentioned enrichment of Ena and Pma transporters the number of other genes does not differ from the numbers of genes found in the less halotolerant *W. sebi*. *Wallemia ichthyophaga* therefore appears to be using a different salt-combating strategy then the extremely halotolerant ascomycete *H. werneckii*. A recent genome analysis in that case revealed significantly increased numbers of most of the alkali cation transporters [37]. For example, *H. werneckii* contains eight inward K^+^ (Trk) transporters and four outward (Tok) K^+^ channels (only one Trk and no Tok homologues are found in *W. ichthyophaga*), eight Nha Na^+^(K^+^) proton antiporters (two in *W. ichthyophaga*), and six Pho89 Na^+^/P_i_ symporters (only one in *W. ichthyophaga*) [37].

In addition to passive K^+^ channels active processes for K^+^ import may be beneficial in hypersaline environments. These can be carried out by K^+^(Na^+^)-ATPase (alkali cation uptake, Acu, transporters) or K^+^-H^+^symporter (Hak symporters) [36,38]. While no Hak homologues are identified in *W. ichthyophaga*, there are two possible homologues of the otherwise rare Acu ATPases [GenBank:EOQ99826, GenBank:EOR03958], one of which has a clearly recognisable P-type ATPase PF00690 domain.

Intracellular transporters comprise mainly membrane alkali metal cation/ H^+^ antiporters of the vacuole (Vnx1), endosomes (Nhx1), and Golgi apparatus (Kha1), and also a mitochondrial membrane crucial K^+^/H^+^ antiport exchange mechanism (Mdm38 or Mrs7) [19]. All of these intracellular transporters have also been identified in the *W. ichthyophaga* proteome, with all at one copy number except for Kha1, which is present as two copies.

Low numbers of transporters in the proteome of *W. ichthyophaga* and the absence of specific transporters may be connected to life at constant (albeit high) salinity. The absence of, for example, the outward K^+^ channel Tok, may be harmless if the organism is not exposed to severe hypoosmotic shocks and the subsequent need to quickly release the surplus K^+^. Furthermore, continuous removal and/or compartmentalization of sodium in these conditions might not even be feasible due to the high ATP demand. The opposite is true for *H. werneckii*, which can efficiently adapt to a whole range of salinities and contains an abundance of both inward and outward K^+^ channels.

### Other transporters

In contrast to the alkali-metal ion transporters, there are statistically significant contractions for several proteins that are involved in transport of other molecules (Figure 4C-E). Major facilitator superfamily transporters (Figure 4C) are the largest family of secondary transporters, with their wide substrate specificity ranging from ions to carbohydrates, lipids, amino acids and peptides, nucleosides and other molecules [39]. Amino-acid permeases and transporter proteins (Figure 4D) facilitate the cellular uptake of amino acids. Finally, ATP binding cassette (ABC) transporters (Figure 4E) are one of the largest protein superfamilies [40]. Both the ABC and the major facilitator superfamily transporters have been extensively studied due to their roles in the development of multidrug resistance in fungal pathogens and tumour cells. Together, they account for approximately half of all of the genes that encode transporters in fungal genomes [41]. The contraction of both families might indicate that in its environment, *W. ichthyophaga* experiences reduced need for this type of defence against internally produced or external toxins. This latter might be caused by the limited competition with other species, due to the extreme salinity conditions that are preferred by *W. ichthyophaga*. On the other hand, the structure and composition of its unusually thick cell wall that is formed as a response to the high salt concentrations might limit the diffusion of problematic compounds before they even reach the plasma membrane. However, proteins of both superfamilies also have numerous functions other than the export of toxins [41]. Their loss may thus be the consequence of other factors as well, such as little need to export secondary metabolites or to sequester heavy metals, nutritional specialisation for a limited number of substrates or even avoidance of accidental export of osmoprotectants by transporters with broad specificity.

### Genes involved in management of compatible solutes

*W. ichthyophaga* contains genes for several proteins involved in the synthesis and accumulation of the compatible solutes. All of them except Gpp (glycerol-3-phosphatase) are present in more then one copy. Key enzymes for the biosynthesis of three polyols, which are present in *W. ichthyophaga* cells (Zajc et al., unpublished data) were identified: glycerol, arabitol and mannitol. Glycerol is synthesized from dihydroxyacetone phosphate, glycolitic intermediate, via two reaction steps catalyzed by (NAD)-dependent glycerol-3-phosphate dehydrogenase (Gpd) and glycerol-3-phosphatase (Gpp) [42]. As previously shown *W. ichthyophaga* contains a GPD1 homologue, WiGPD1 [EMBL:FR686467, GenBank:EOR01876], the expression of which is salt-induced [11]. A second homologue was found by searching the genome [GenBank: EOR02702]. Expectedly, Gpp [GenBank:EOR02702] and both copies of Gpd are well conserved.

The synthesis of D-mannitol could be perfomed from fructose via a reduction step catalyzed by two NADP-dependent mannitol dehydrogenases [GenBank:EOR01463, GenBank:EOQ99146] as was previously described also for the basidiomycete *Agaricus bisporus*[43]. In fungi arabitol is produced from D-ribulose-5-phosphate, an intermediate of pentose phosphate pathway, by two D-arabinitol-2-dehydrogenases (homologues in *W. ichthyophaga* are [GenBank:EOR02008, GenBank:EOQ98686]). Homologues of these enzymes were identified also in *W. sebi* (in even more copies): four mannitol dehydrogenases and three D-arabinitol-2-dehydrogenases.

When the cells of *S. cerevisiae* are subjected to hyperosmotic shock, leaking of glycerol is counteracted by active import. This is performed by glycerol/ proton symporters of the plasma membrane, Stl1 [44]. In these conditions the aquaglyceroporin channel Fps1 is closed. It opens during a hypoosmotic shock and thus allows quick glycerol expulsion [45]. In *W. ichthyophaga* four homologues of Stl1 were found [GenBank:EOR04223, GenBank:EOQ99617, GenBank:EOR01226, GenBank:EOR01982], as well as three aquaglyceroporin related proteins [GenBank:EOR00599, GenBank:EOR04990, GenBank:EOQ99141]. *W. sebi* contains the same number of homologues, all of them highly similar to those of *W. ichthyophaga*.

### Mating and meiosis genes

In organisms that show no observable behavioural and morphological traits that are characteristic of sexual reproduction, searching for the mating and meiosis homologues in the genome can shed some light on their reproductive cycles [46]. In basidomycetous fungi sexual reproduction is genetically governed by a tetrapolar *MAT* locus with pheromone/ pheromone receptors and homeodomain (HD)-containing transcription factors encoded by two unlinked loci. The *MAT* loci have expanded in some cases, and in others they have fused, which results in a tetrapolar-to-bipolar transition [47].

BLASTp searches of homeodomain protein sequences from different basidiomycetous fungi in the proteome of *W. ichthyophaga* identified five genes that encode putative HD-motif transcription factors located on different scaffolds [GenBank:EOR04835, GenBank:EOR03936, GenBank:EOR03899, GenBank:EOR01329, GenBank:EOQ99399]. Only one of these, EOR03899 (hypothetical WiSxi1) was similar (e-value, 10^-5^) to the HD genes involved in mating in basidiomycetes, as shown by the comparison of the *W. ichthyophaga* HD proteins against GenBank. It [GenBank:EOR03899] is most similar to Sxi1D alpha from *Cryptococcus neoformans var. neoformans* [GenBank:ABR67867]. Together with Sxi2a, Sxi1D is required for the initiation of dikaryon formation in *C. neoformans*[48]. In addition, BLASTp searches for pheromone response factors reveal three putative mating-related DNA-binding proteins with high-mobility group (HMG-box) domains [GenBank:EOR04027, GenBank:EOQ98813, GenBank:EOQ98831]. All three of these are somewhat similar to basidiomyceteous HMG-box transcription factors (most similar to the HMG-box transcription factor from *Ustilago hordei* [GenBank:CCF49140]; e-value approx. 10^-13^). No discernible *MAT* locus was identified. Furthermore, no pheromone receptors or pheromone precursors were found.

The results of the investigation of the proteome of *W. ichthyophaga* for the meiosis-specific genes [49] also suggest that *W. ichthyophaga* cannot outcross. Only three (putative Dmc1, Mer3 and Msh4; e-values, <10^-40^) out of eight meiosis-specific homologues were identified in *W. ichthyophaga*, meaning that it does not have a complete set of meiotic machinery. Since the presence of a representative set of meiotic genes provides strong inferences about meiosis and sex [46], it appears that *W. ichthyophaga* is incapable of sexual reproduction. This is in agreement with the fact that there are no reports on sexual reproduction for this species in the existing literature.

Contrary to this, in the closely related *W. sebi* the two mating-type genes are located near to each other (Sxi1 and Ste3 pheromone receptor homologue; ~20 kb apart) [4]. The inspection of the region between these two genes identified two other putative mating-type genes; a pheromone and a transcription factor that encodes a HMG DNA-binding motif. In addition, the genome of *W. sebi* contains a near-complete set of meiosis genes, which lacks only the homologue of Hop1. Therefore, it appears that *W. sebi* is capable of sexual reproduction, although this part of its life cycle remains cryptic. It appears to have a bipolar mating system with two mating types, analogous to some other Basidiomycota [4].

The lack of sexual reproduction in *W. ichthyophaga* is not entirely surprising*.* In stable extreme environments, asexual reproduction might be advantageous, as it avoids the energy expenditure needed for producing gametes and attractants. Strict asexuality in specialised and adapted local populations would also help in the preservation of well-adapted genomic configurations [50]. Without sexuality, genetic drift can rapidly fix alleles in fragmented small populations that are adapted to the extreme habitats. These might all contribute to the mainly mitotic life style that many extremophilic fungal species have [9].

### Transcriptome of *Wallemia ichthyophaga* at limiting salinities

Mapping more than 50 million EST sequences to the *W. ichthyophaga* genome aligned 83.96% and 83.70% of reads (transcriptome grown at 10% and 30% NaCl (w/v), respectively; Additional file 4: Table S3). Of these, over 99% matched to unique locations in the genome. At each salinity, approximately 95% of the predicted genes were more than 90% covered by the ESTs (Additional file 1: Figure S3), which indicates the high quality of the sequencing and mapping.

**Figure 6:**
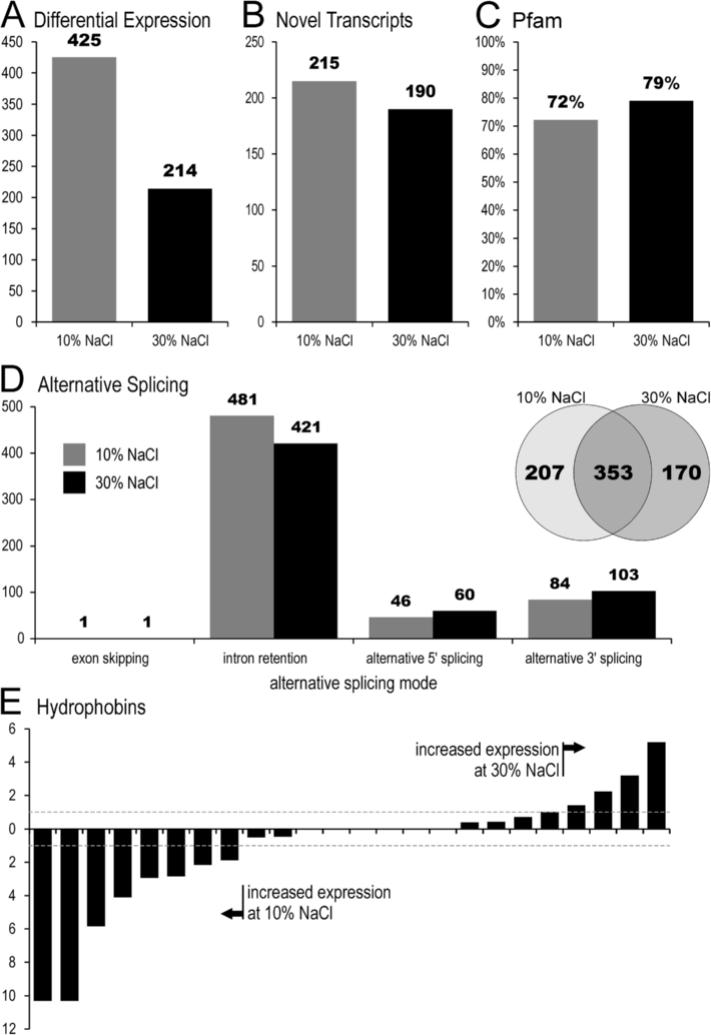
**Transcriptome of *Wallemia ichthyophaga *at 10% NaCl and 30% NaCl (w/v). A**. Number of genes with increased expression at each salinity, compared to the other salinity. **B**. Number of novel transcripts predicted at each salinity. Gene models at least 200 bp away from known upstream or downstream genes were considered as novel transcripts. **C**. Proportion of proteins with increased expression at each salinity that were matched to at least one Pfam family. **D**. Number of genes with different alternative splicing modes at each salinity. Two intersecting circles show the number of genes with alternative splicing observed only at 10% NaCl, only at 30% NaCl, or at both (middle). **E**. Differences in expression of hydrophobins between the two salinities. Each bar represents a log_2_ ratio of expression at each salinity for an individual hydrophobin. All values above and below the dashed grey lines were considered as significantly differentially expressed.

Changes in salinity have been reported to trigger drastic remodelling of the transcriptome of *S. cerevisiae*. However, many of these changes are only transient, and they soon return to near basal levels [51], which indicates that they are not involved in long-term survival at high salt concentrations. On the other hand cDNA subtraction analysis of *H. werneckii* cells adapted to 17% and 25% NaCl revealed a long-term differential expression of 95 genes. More then one third of them were shown to interact with the high osmolarity glycerol pathway [52]. Our purpose was therefore not to investigate the temporary transcriptional perturbations of *W. ichthyophaga* under osmotic stress, but the transcriptome differences in cells growing at the lower and upper salinity limits that are tolerated by *W. ichthyophaga*. Of the total 4884 genes in *W. ichthyophaga*, 639 (13.1%) were differentially expressed when compared across the transcriptomes of cells grown at these salinities. The number of genes with increased expression was twice as high (425) at lower salinity compared to higher salinity (214; Figure 6A and Additional file 5: Table S4). Of these, 72% and 79% (low and high salinity, respectively) were matched to at least one Pfam family (Figure 6C). Around 200 novel transcripts were predicted at each salinity (Figure 6B). Twelve KEGG pathways had significantly higher proportions of differentially expressed genes than expected (Additional file 6: Table S5).

Alternative splicing was observed for 730 genes (15.0%), and for 51.6% of these, this occurred only at one of the limiting salinities. The majority of cases were identified as intron retentions, followed by alternative 3’, and then 5’, splicing (Figure 6D). Only one instance of exon skipping was detected. Such a distribution of alternative splicing modes is in agreement with observations from other fungi [23], where alternative splicing levels are generally much lower than in plant or human cells. While it is not possible to directly compare the values observed in other species [53], the around 700 events observed in *W. ichthyophaga* at each salinity was not outstanding (Additional file 1: Figure S4). Nevertheless, due to the small number of genes, the number of alternative splicing events per gene is close to the highest values observed in other species [23].

Transmembrane alkali-metal cation transporters are among the most important proteins in hypersaline environments [19]. Although hardly any studies have been performed on cells grown at constant salinities rather than those exposed to salinity shock, it has been noted that in *S. cerevisiae*, some groups of metal-cation transporters are expressed constitutively, while others have complex salt-responsive transcriptional regulation (e.g., P-type sodium ATPase Ena1 [19]). In the halotolerant ascomycetes *H. werneckii* and *Debaryomyces hansenii*, the expression of P-type H^+^ and Na^+^ ATPases is salt dependent, even when adapted to constant salinity [37,54,55]. It was therefore unexpected that the expression of most of the genes encoding metal-cation transporters was not affected by growth at 10% NaCl or 30% NaCl (w/v). Only three genes fulfilled the criteria for differential expression: [GenBank:EOR00619, GenBank:EOR04078, GenBank:EOR03958].

The first of these, EOR00619, was more expressed at high salinity (log_2_ ratio of expressions, 1.52), and it is a putative Na^+^/ phosphate symporter (homologue of Pho89). Pho89 is the only Na^+^-coupled secondary anion transport system in *S. cerevisiae*, and it is strongly induced at alkaline pH [56]. Since in such environment the use of a transmembrane proton gradient to drive secondary transporters is hindered, Na + gradient could serve as an alternative source of energy. The possible role of this transporter in hypersaline conditions was also noted in *H. werneckii*, which contains six copies of its gene [37]. Our results might indicate that *W. ichthyophaga* cells use this energy even under conditions of low pH, since the pH in the laboratory medium falls below 4 already in the exponential growth phase. The expression of [GenBank:EOR04078] (a putative P-type Na^+^ ATPase), and [GenBank:EOR03958] (a P-type ATPase of unknown specificity, possibly an Acu K^+^ importer), was higher at low salinity (log_2_ ratios, 1.00, 1.27, respectively). While the differences in expression are relatively small, these findings are difficult to explain.

All of the other transporters remained unaffected by the difference in salinity. Furthermore, their expression was relatively low. For example, when genes were ordered by their expression at high salinity, the two Na^+^-exporting P-type ATPases were in positions 3812 and 4613 of the total of 4884 predicted proteins, while two Na^+^/H^+^ antiporters were at 2075 and 3927 (Additional file 5: Table S4). Among the first 400 of the most-expressed proteins, there were only various subunits of the P-type H^+^-transporting ATPase, and a putative arsenite transporter [GenBank:EIM21938]. None of these were differentially expressed.

It has to be noted that the sequencing of the transcriptome will only reveal regulation at the level of transcription and mRNA stability. This cannot provide any indication about other possible modes of posttranscriptional control. Additionally, the functions of the genes analysed might be complemented by unconventional transporters that have not yet been identified. On the other hand, the apparent non-responsiveness of transporters might be just one of the aspects of the ecological strategy of *W. ichthyophaga*, a species that has evolved into a unique example of a narrowly specialised fungal halophile [9]. The observed absence of transcriptional response might explain the unusual inability of *W. ichthyophaga* to grow without salt.

Nevertheless, the transcription of some other genes responds to changes in salinity, among them also the above discussed hydrophobins. Half of these genes are differentially expressed (Figure 6E). It is interesting to note that some of these are more expressed at high salinity, and some at low salinity. Furthermore, there was no association between their expression profiles and isoelectric points, or the numbers of acidic amino acids. As was previously noted, the presence of multiple hydrophobin genes in an organism might be due to their different expression profiles at different developmental stages or under different environmental conditions, or to their different functional roles that are reflected in structural differences [26].

Among the most differentially expressed at low salinity, with log_2_ ratios of −9.19 and −3.34, were two expansin-like proteins of *W. ichthyophaga* [GenBank:EOR02784, GenBank:EOR03994]. These genes are distantly related to the plant expansins, through the presence of the double-psi beta-barrel domain and signal peptide [57]. Expansins loosen the cell wall by disrupting the non-covalent bonds between cellulose microfibrils and matrix polymers through a non-enzymatic mechanism [58]. This activity was recently shown also for a fungal expansin-like protein from *Bjerkandera adusta*[57]. As hydrophobins these proteins may also be linked to the cell wall and possibly play a role in major changes in the cell wall of *W. ichthyophaga* at different salinities, which were mentioned earlier.

Thirty-four genes showed both high expression (RPKM >300 for at least one salinity, with RPKM indicating the number of reads which map per kilobase of the exon model per million mapped reads) and large differences in expression between the two salinities (absolute log_2_ ratio >2; Additional file 7: Table S6). Eight proteins with higher expression at high salinity included enzymes involved in the degradation of lipids ([GenBank:EOQ99895], a lipase) and their use in gluconeogenesis ([GenBank:EOR01390], an isocitrate lyase that enables synthesis of glucose from acetyl-CoA; and EOR04235, phosphoenolpyruvate carboxykinase, which catalyses the rate-limiting step of gluconeogenesis). In contrast at the lower salinity there was a higher expression of a phosphoglycerate mutase-like protein ([GenBank:EOR03927], involved in the eighth step of glycolysis), glycoside hydrolase [GenBank:EOQ98927], and a protein highly similar to fatty-acyl-CoA synthase from *W. sebi* [GenBank:EOQ99675]. This possibly indicates that at lower salinity, the metabolism is directed from carbohydrates to lipids, while the opposite is true at higher salinity. Among other genes of interest is an elevated expression of a stress-response protein, Rds1 [GenBank:EOR00712] at high salinty and of a tyrosinase-like protein [GenBank:EOR01004] at low salinity. The latter is one of three homologues in the genome (in addition to [GenBank:EOR00580, GenBank:EOR00979]). Tyrosinases are involved in melanin synthesis, a stress-protective pigment, which can also play a role in adaptation to hypersaline environment [59]. Nevertheless, it has to be noted that no melanin pigments have been reported to date in *Wallemia* spp., only a number of different pyrrolylpolyenes [60].

A list of all differentially expressed genes is available in Additional file 7: Table S6.

## Conclusions

It is believed that low levels of adaptability and genetic recombination are important in the survival strategy of *W. ichthyophaga*[9]. The extreme specialisation of *W. ichthyophaga* indicates that it has adapted to a relatively stable hypersaline environment where competition from other species is scarce. This allows *W. ichthyophaga* to survive despite its relatively long generation times, its inability to grow without salt, and its lack of sexual reproduction. The characteristics of its genome and its transcriptomic response to salt confirm these findings. The genome and the number of predicted genes are among the smallest observed for fungi. Intriguingly, analysis of the genome also shows that it is possible to survive and grow in solutions saturated with NaCl with relatively small numbers of ion-transporter genes and although their transcription is relatively low and non-responsive to different salt concentrations.

A long-term survival strategy, where persistence is more important than rapid reproduction, good adaptability or competition for resources, will favour energy-efficient passive barriers against harmful effects of high salt concentrations. Previous morphological studies have reported the unusually thick cell wall of *W. ichthyophaga*, which could be one such mechanism [8]. Hydrophobins, proteins with multiple cellular functions, might also have important roles in fortifying the cell wall of *W. ichthyophaga* against the hostile environment, as indicated by genomic and transcriptomic analyses. This is especially so, as their unusually high proportion of acidic amino acids is a phenomenon that is a known characteristic of halophilic proteins from other organisms.

The peculiar lifestyle of *W. ichthyophaga* has had ample time to evolve. According to our estimates, 250 million years have passed since the ancestor of Wallemiomycetes separated from the ancestor of the contemporary Agaricomycotina, the closest known relative of *Wallemia* spp. The distinct characteristics of *W. ichthyophaga* make it a very interesting organism for studies of adaptation to hypersaline environments, which are different from those that have evolved in other species. The availability of its genomic sequence should be of significant help in such studies. At the same time, as shown with the findings of the unusual hydrophobins, it can also lead to the discovery of molecules that evolved along different evolutionary trajectory than their homologues from other species, and thus have novel traits that might be of interest in biotechnology and other fields.

## Methods

### Strain and DNA/RNA preparation

The *W. icththyophaga* (type strain EXF-994) used in this study was isolated from extremely saline water of Sečovlje solar saltern (Adriatic coast, Slovenia) and is preserved in the culture collections of the Department of Biology, Biotechnical Faculty, University of Ljubljana (EXF). The cells of *W. ichthyophaga* were grown at 24°C on a rotary shaker (180 rpm) in defined YNB medium (ForMedium, UK): 0.17% (w/v) yeast nitrogen base, 0.08% (w/v) complete supplement mixture (both Qbiogene), 0.5% (w/v) ammonium sulphate, 2.0% (w/v) glucose, in deionised water, with pH adjusted to 7.0 and supplemented with NaCl to 10%, 20% and 30% NaCl (w/v).

Growth was monitored by measuring the pH of the medium. The cells of mid-exponential cultures (pH 4.0) were harvested by centrifugation (4000× *g*; 10 min), frozen in liquid nitrogen, and homogenised using a pestle and mortar.

Highly purified fungal genomic DNA was isolated from mid-exponential cells grown in YNB media with 20% (w/v) NaCl, using the phenol/ chloroform/ isoamyl alcohol method, modified for DNA isolation from filamentous fungi, as described previously [61]. The quality and quantity of the DNA was evaluated on standard 1% agarose gel electrophoresis, as well as spectrophotometrically with NanoDrop 2000 (Thermo Fisher Scientific, USA).

The RNA of the *W. ichthyophaga* cells grown at 10% and 30% NaCl was isolated using the TRI Reagent (Sigma-Aldrich, Germany), according to the manufacturer instructions. Possible DNA contamination was degraded with DNAse I (Thermo Fisher Scientific - Fermentas, Lithuania), and the integrity and purity of the RNA was evaluated spectrophotometrically and by capillary electrophoresis (Agilent 2100 Bioanalyser; Agilent Technologies, USA).

### Genome sequencing and assembly

From the genomic DNA of *W. ichthyophaga*, 500 bp and 2000 bp DNA sequencing libraries were constructed using 10 μg and 20 μg DNA, respectively. A total of 1.89 Gb and 1.80 Gb reads were generated by Illumina Hiseq™ 2000 at BGI-Shenzhen (Shenzhen, China). To ensure the accuracy of assembly, reads with 40 low-quality (≤Q2) bases, or 10% Ns, or 15 bp overlap between adapter and duplications were filtered. The short reads from the two libraries were assembled by *SOAPdenovo* 1.04 [62,63], with optimal assembly acquired with the key parameter K = 21.

### Gene prediction and annotation

The prediction of the genes was made by determining the putative open reading frames with GeneMark-ES 2.3e [64]. Repeat sequences were identified by Repeat Masker version 3.3.0 with Repbase version 15.08, and the following parameters: -no_is, -norna, -engine, -s, -parallel = 1; and Repeat Protein Mask with parameters: -noLowSimple, -pvalue = 1e-4 [65]. Tandem repeats were found using the Tandem Repeat Finder software 4.04 [66]. Non-coding RNA was predicted by rRNAmmer 1.2, tRNAscan-SE 1.23, and Rfam 10.1. The protein-encoding genes were annotated through BLASTp searches in the KEGG (release: 55.1 2010-09-01) and COG (release: 20090331) databases, at the threshold e-value ≤1× e^-10^, and the best hit was filtered using a 50% identity cut-off value.

### cDNA Library construction, and sequencing

The cDNA library was constructed using 40 μg RNA for each of the two salinity samples. In short, beads with oligo(dT) were used to enrich poly(A) mRNA, which was then disrupted into short fragments of 200 nt to 700 nt. These were used as first-strand cDNA templates synthesised by using random hexamer primer. The second-strand cDNA was synthesised by adding buffer, dNTPs, RNaseH and DNA polymerase I. The cDNA library was purified using QiaQuick PCR extraction kits and resolved in elution buffer for end repair and adding poly(A). Finally, cDNA fragments were ligated with sequencing adaptors and fragments of 200 bp (±25 bp) were selected for the PCR amplification. The two constructed cDNA libraries were sequenced by Illumina Hiseq™ 2000 at BGI-Shenzhen (Shenzhen, China).

### Trancriptome data processing, alternative splicing and novel transcript predictions

Raw data generated by the sequencer were converted to raw nucleotide reads with Illumina GA Pipeline 1.6. The clean reads were acquired by the removal of the adaptor and the low quality reads (Q ≤5), and they were mapped to the genome and gene sequences of *W. ichthyophaga.* This was done using the SOAPaligner/soap2 2.20 [67]. Up to five base mismatches were allowed.

The TopHat read-mapping algorithm [68] that does not rely on known splice sites was used to find splice junction sites of transcripts. This provided the information relating to the combinations of different exons of the individual transcripts, so that four basic types of alternative splicing events were distinguished (exon skipping, intron retention, alternative 5’ splicing, and alternative 3’ splicing). Candidates for novel transcripts were assigned all of the gene models in intergenic regions from 200 bp upstream or downstream from a gene with a length >150 bp and an average coverage >2.

### Graphical representation of the genome and comparison with *Wallemia sebi*

Graphical representation of the genome was constructed with the Circos software version 0.62 [69]. The GC content was calculated in 1000 bp windows with gccount from Control-FREEC package 5.9 [70]. Repetitive sequences were identified with RepeatMasker 3.3.0 [71] with Fungi used as the model for analysis. Gene duplications were detected by aligning predicted proteins back to the genome with Exonerate 2.2.0 using the protein2genome model [72] and limiting the reported hits to those above the 50% maximal score obtainable for that query. The number of hits was counted for each query.

The whole genome alignment between *W. ichthyophaga* and the publicly available genome of *W. sebi*[4] was calculated with the promer algorithm as implemented in Mummer 3.23 and plotted with mummerplot utility [73]. All parameters were the same as described in [74] except that instead of discarding scaffolds less than 500 kbp only scaffolds less than 200 kbp were discarded.

The numbers of shared and unique proteins of *W. ichthyophaga* and *W. sebi* were determined by an all-against-all blast of their whole proteomes (e-value cut-off, 10^-6^).

### Phylogenetic analysis

A super alignment of the fungal proteomes was constructed with the Hal pipeline [75], allowing for no missing data. As well as *W. ichthyophaga* and *W. sebi*[4], several other publicly available proteomes were included. The following were obtained from the Broad Institute of MIT and Harvard (http://www.broad.mit.edu): *Coprinopsis cinerea* (*Coprinopsis cinerea* Sequencing Project); *Cryptococcus neoformans* (*Cryptococcus neoformans var. grubii* H99 Sequencing Project); *Ustilago maydis* (*Ustilago maydis* Sequencing Project); *Puccinia graminis* (*Puccinia* Group Sequencing Project); *Candida albicans* (*Candida* Sequencing Project); *Aspergillus nidulans* (*Aspergillus* Comparative Sequencing Project); *Neurospora crassa* (*Neurospora crassa* Sequencing Project); *Rhizopus oryzae* (*Rhizopus oryzae* Sequencing Project). The other proteomes were *Saccharomyces cerevisiae* (SGD project. http://www.yeastgenome.org/download-data/ (28.1.2013)); *Rhodotorula graminis*[76]; *Agaricus bisporus*[76]; and *Malassezia globosa* (Procter & Gamble: http://www.pgbeautygroomingscience.com/dandruff-genome.html/ (28.1.2013).

Conservative alignment (118776 bp) was used to estimate the best protein evolution model with ProtTest 3.2.1 [77]. The species tree was generated with the PhyML 3.0 software [78] with aLRT implementation, for the calculation of branch supports as Chi2 based support. The analysis was run using the LG model of evolution. The ProtTest estimate of the alpha parameter of the gamma distribution of six substitution rate categories (1.136) and the determined proportion of invariable sites (0.094) were used. The tree was then calibrated with r8s software [79], using four previously published calibration points [24]: *Rhizopus oryzae*–Dykaria split 495 mya, Ascomycota–Basidiomycota split 452 mya, Pezizomycotina crown 215 mya, Basidomycota crown 340 mya.

Protein sequences containing the Pfam domain PF00690 (P-type ATPase) were aligned using the L-INS-i method in the MAFFT software [80]. The gene tree was generated with the PhyML 3.0 software [78] with aLRT implementation, for the calculation of branch supports as Chi2 based support. The LG model of protein evolution was used, together with the alpha parameter of gamma distribution of six substitution rate categories (0.988) and the determined proportion of invariable sites (0.008), as estimated by ProtTest 3.2.1 [77]. A second tree (for comparison purposes, not shown) was generated by applying a maximum parsimony method as implemented in the Mega software version 5.05 [81].

### Evolution of protein families

Analysis of protein family expansions and contractions was performed with the CAFE software [82]. Pfam domains of selected fungal proteomes were identified with a stand-alone Pfam scanner and a database downloaded on 30.1.2013 [83]. This was used to produce a Table of Pfam domains, which was used as input of CAFE, together with the chronogram constructed from the whole proteomes, as described above.

Proteins containing the hydrophobin Pfam domain (PF01185.13) were aligned using the L-INS-i method in the MAFFT software [80]. Sequence logos of amino-acid residues were drawn using the WebLogo 3 service [84], after removing the positions that were present in less than 75% of the proteins of a given species. The properties of the proteins were calculated with the pepstats utility included in the EMBOSS suite [85].

### Searching for mating- and meiosis-related proteins and alkali-metal cation transporter homologues

To investigate the molecular evidence of sex in *W. ichthyophaga*, we searched for the presence of mating-type and meiotic-protein homologues in the proteome. Databases of mating-related proteins, homeodomain-containing proteins (PF00046), pheromone factor receptors (PF02076), pheromone response factors (Prf1), and pheromone precursors (PF08015) were created by retrieving sequences of selected basidiomycetes (*Agaricus bisporus*, *Coprinopsis cinerea, Cryptococcus neoformans, Cryptococcus heveanensis, Puccinia graminis, Ustilago maydis, and W. sebi*) and *S. cerevisiae* from UniProt Consortium (http://www.uniprot.org/). These were used as queries in BLASTp for the investigation of the mating-type gene homologues in *W. ichthyophaga.* The results were filtered according to the e-value cut-off 10^-2^ criteria, and compared against GenBank. Furthermore, the genome of *W. ichthyophaga* was investigated for the representative set of meiotic genes (i.e., a ‘meiosis detection toolkit’) [49], using protein homologues from *S. cerevisiae* and *C. cinerea*[86].

In the same way, the identification of all alkali-metal cation transporters encoded in the *W. ichthyophaga* genome was performed. In short, a database of *S. cerevisiae* transporters (Trk1, Trk2, Tok1, Pho89, Nha1, Ena and Pma P-type ATPases, Kha1, Nhx1, Vnx1, Pmc1, Mrs7) and transporters identified in other fungi (Acu1-4, Hak1-4) (*Ajellomyces capsulata, Ajellomyces dermatitidis, Candida albicans, Candida dubliniensis, Debaryomyces hansenii, Hordeum vulgare, Magnaporte oryzae, Millerozyma farinosa, Neosartorya fumigata, Neurospora tetrasperma, Physcomitrella patens, Pichia angusta, Sporisorium reilianum, Schwanniomyces occidentalis, Ustilago maydis, Ustilago hordei, and Wickerhamomyces ciferrii*) was constructed by collecting the protein sequences from UniProt Consortium. BLASTp results were filtered according to the e-value cut-off 10^-40^, and compared against GenBank.

## Competing interests

The authors declare that they have no competing interests.

## Authors’ contributions

JZ isolated the DNA and RNA, analysed the mating-type and meiosis-related genes, genes encoding metal-cation transporters and hydrophobins, and the CAFE output, interpreted part of the transcriptomic data, and participated in writing the manuscript. YL submitted the data to GenBank and participated in writing the Methods section of the manuscript. WD, ZY and JH performed the sequencing, assembly and annotation of the genome and transcriptome, and participated in the project coordination. CG constructed the Circos images, performed the phylogenetic and CAFE analyses, interpreted part of the transcriptomic data, and participated in writing the manuscript. NGC conceived the study and participated in its coordination. All authors have read and approved the final manuscript.

## Supplementary Material

Additional file 1: Figure S1 Classification of the predicted genes into the KEGG database categories. **Figure S2.** Classification of the predicted genes into clusters of orthologous groups (COG database). **Figure S3.** Distribution of transcriptome gene coverage. **Figure S4.** Number of different alternative splicing events at each salinity. Click here for file

Additional file 2: Table S1 Number of proteins of *W. ichthyophaga* with the given Pfam domain. Click here for file

Additional file 3: Table S2 Evolution of protein families. Results of the analysis with the CAFE software. For each family, the P values for the family expansion/ contraction are shown for the whole tree, as well as for the branches leading to *W. ichthyophaga*, *W. sebi*, both *Wallemia* spp., and Agaricomycotina. Only families with a family-wide P-value lower than 0.01 are shown. Click here for file

Additional file 4: Table S3 Alignment statistics of the transcriptome of *W. ichthyophaga* at 10% and 30% NaCl (w/v). Click here for file

Additional file 5: Table S4 Expression of all of the genes of *W. ichthyophaga* at 10% and 30% NaCl (w/v). Click here for file

Additional file 6: Table S5 KEGG database pathways with a significantly higher proportion of differentially expressed genes than expected (when comparing transcriptomes at 10% and 30% NaCl [w/v]). Click here for file

Additional file 7: Table S6 Differentially expressed genes of *W. ichthyophaga* at 10% and 30% NaCl (w/v). Click here for file
