# Supplementary material for: Genome and transcriptome sequencing of the halophilic fungus Wallemia ichthyophaga: haloadaptations present and absent
Source: BMC Genomics. 2013 Sep 13;14:617. doi: 10.1186/1471-2164-14-617 (PMC3849046; doi:10.1186/1471-2164-14-617)
Supplement: Additional file 1: Figure S1 — Classification of the predicted genes into the KEGG database categories. Figure S2. Classification of the predicted genes into clusters of orthologous groups (COG database). Figure S3. Distribution of transcriptome gene coverage. Figure S4. Number of different alternative splicing events at each salinity. [file 1471-2164-14-617-S1.pdf]

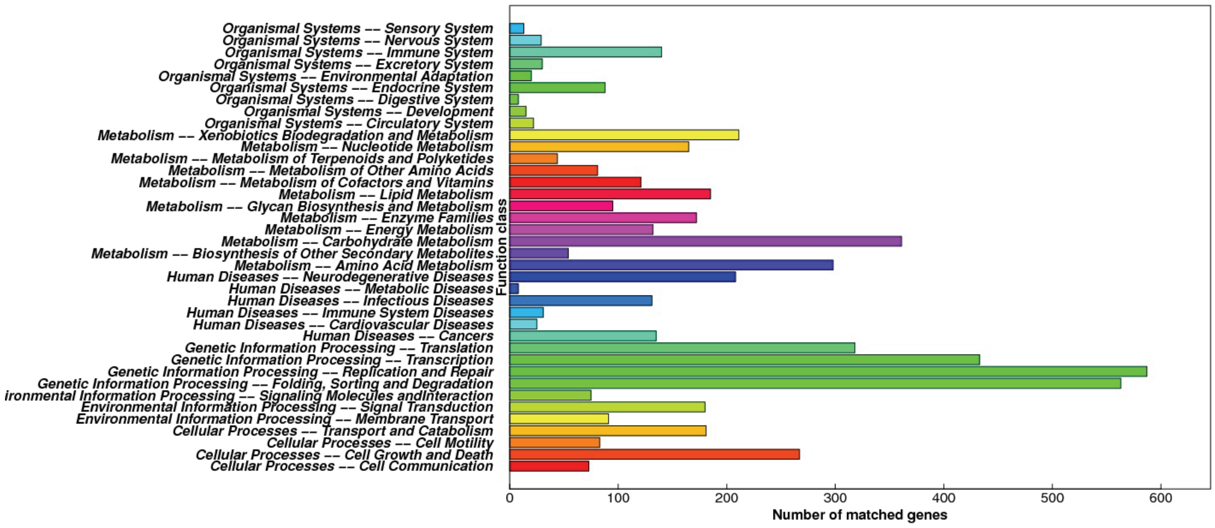

Figure S1 Classification of predicted genes into KEGG categories.

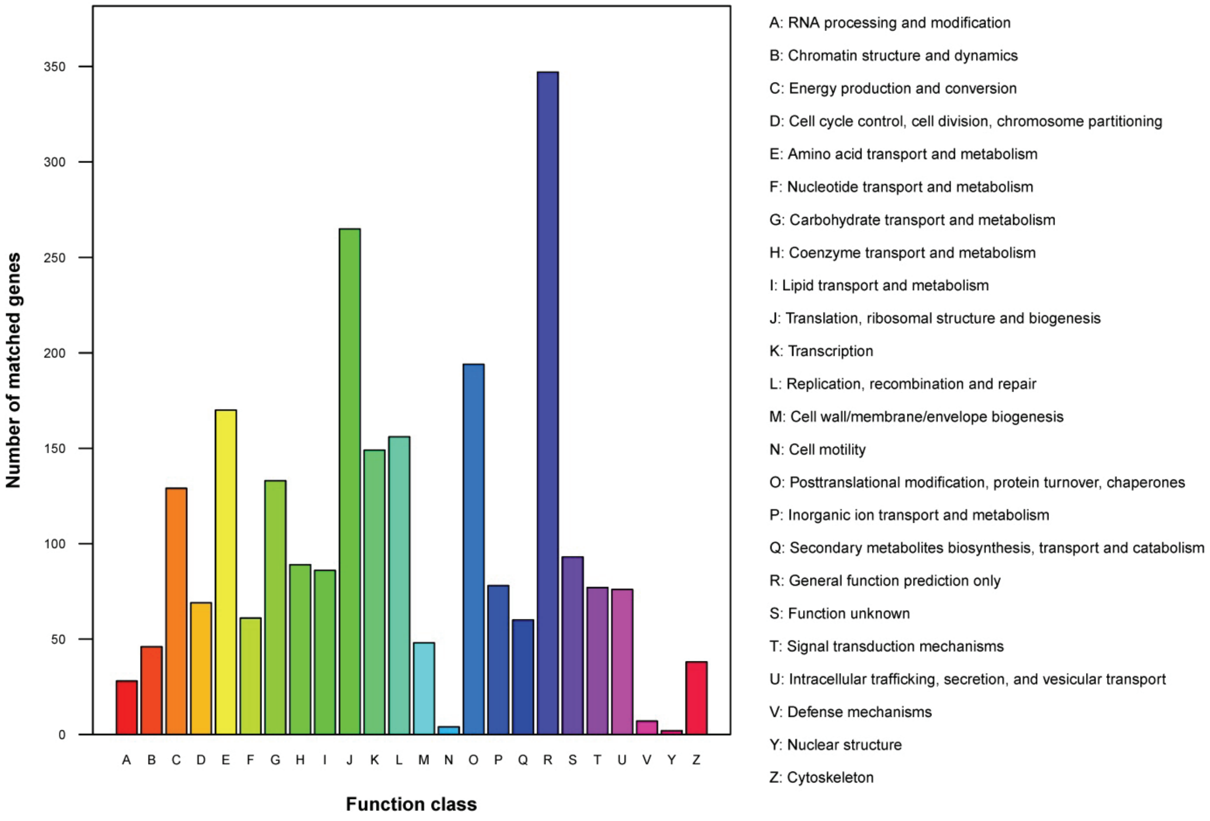

Figure S2 Classification of predicted genes into COG clusters.

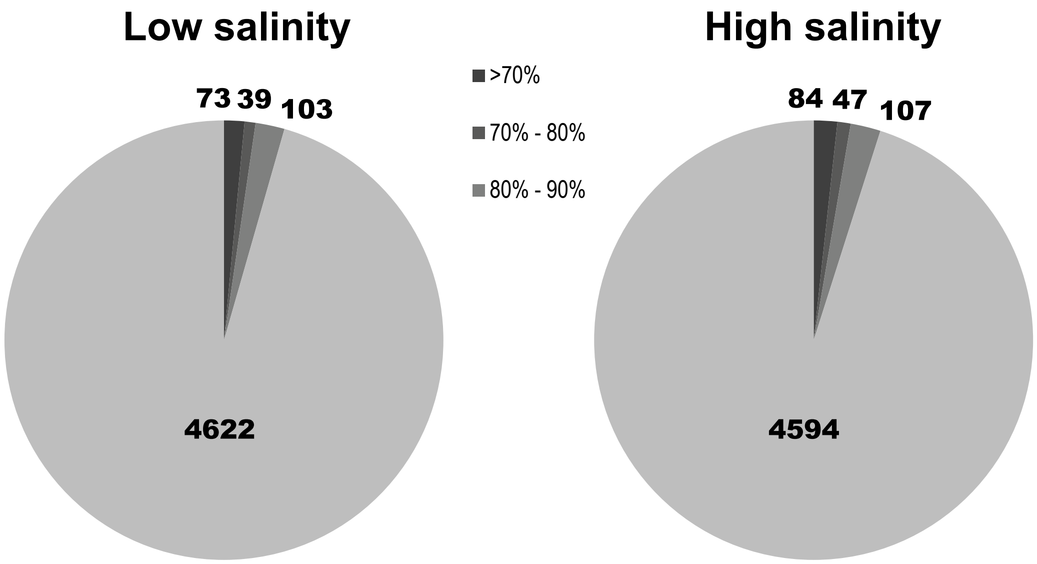

Figure S3 Distribution of transcriptome gene coverage. The pie charts show the distributions of gene coverage (the percentage of a gene covered by reads) of *Wallemia ichthyophaga* grown at 10% NaCl (low salinity) and 30% NaCl (high salinity).

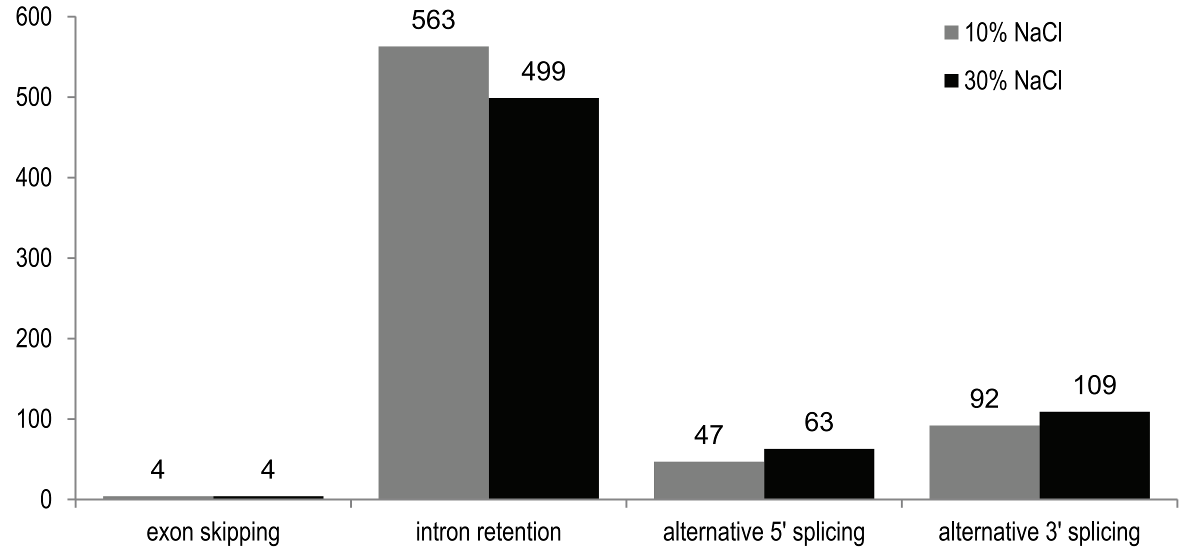

Figure S4 Number of different alternative splicing events at each salinity.
